# Spatial and vertical distribution analysis of heavy metals in urban retention tanks sediments: a case study of Strzyza Stream

**DOI:** 10.1007/s10653-019-00439-8

**Published:** 2019-10-09

**Authors:** N. Nawrot, E. Wojciechowska, K. Matej-Łukowicz, J. Walkusz-Miotk, K. Pazdro

**Affiliations:** 1 Faculty of Civil and Environmental Engineering, Gdansk University of Technology, Narutowicza 11/12, 80-233 Gdańsk, Poland; 2 Marine Geotoxicology Laboratory, Institute of Oceanology of the Polish Academy of Sciences, Powstańców Warszawy 55, 81-712 Sopot, Poland

**Keywords:** Bottom sediments, Heavy metals, Pollution indices, Granulometric analysis, Urban catchment

## Abstract

Concentrations of seven heavy metals (HMs): Zn, Cu, Pb, Cd, Ni, Cr, and Fe in core samples of bottom sediments from four retention tanks (RTs) located along the Strzyza Stream in northern Poland (Gdansk) were measured to obtain a general view of sediment quality. The recognition of contamination and potential environmental impacts is the main aim of this paper. A total of 96 sediment samples were collected from eight sampling sites at depths of 0–2 cm, 8–10 cm, 16–18 cm, 24–26 cm. Concentrations of Cu, Zn, Pb, Fe, Ni, and Cr were measured with AAS while Cd concentration was measured with ICP-MS. Granulometric analyses with normalized sieve apertures were carried out. Geochemical indices: enrichment factor, anthropogenic factor, and modified degree of contamination (mCd) were used to assess the contamination level. Concentrations of HMs in sediments changed in the range: Cu (3.24–119 mg/kg d.w.), Zn (12.5–584 mg/kg d.w.), Pb (4.91–309 mg/kg d.w.), Cd (0.003–0.716 mg/kg d.w.), Ni (1.57–25.8 mg/kg d.w.), Cr (2.45–74.5 mg/kg d.w.), and Fe (3993–63817 mg/kg d.w.). The sequential extraction verified the bonding of HMs with non-mobile fractions. Geochemical indices showed widespread pollution by Cu, Pb, Cd, and Zn. Cluster and factor analysis distinguished three related subgroups of HMs: Pb, Ni–Cr, and Fe–Cd–Cu–Zn, suggesting possible common source of each subgroup. Strongly contaminated sediments were distinguished in sediment cores in two middle stream RTs characterized by intense urbanization in their direct catchments.

**Electronic supplementary material:**

The online version of this article (10.1007/s10653-019-00439-8) contains supplementary material, which is available to authorized users.

## Introduction

Nowadays, focusing on new emerging organic pollutants may lead to a situation when formerly recognized highly toxic pollutants like heavy metals (HMs) are considered to be less important. HM concentrations rise in response to diverse human activities: industry, traffic and simply “living”, as well as being washed out from roof tops with copper and zinc ornaments (Angrill et al. 2017; Charters 2016a, b; Murphy et al. 2015; Nawrot et al. 2018; Nawrot and Wojciechowska 2018; Robertson et al. 2003). Most of Poland’s population still relies on traditional solid fuels for heating, producing high emissions of a number of air pollutants like HMs, CO, SO_2_, particulate matter, polycyclic aromatic hydrocarbons, etc. during combustion (Du et al. 2018). HM presence in soils and sediments cannot be seen as neutral to humans nor to the environment due to their mutagenicity and trophic accumulation (Ergönül and Altindağ 2014). Although some elements, like Zn or Cr, are essential for human, animals and plants health and growth, their enrichment may reach a level which poses a potential health risk (Modabberi and Tashakor 2018). Moreover, some metals, including Cd, Pb, and Hg are abundantly available for the biome and are highly toxic even at low concentrations.

HM pollution of soils and sediments in relation to mining and other industrial sites is often reported (Weissmannová et al. 2015; Guo et al. 2005; Hansen et al. 2012; Ji et al. 2018; Jinmei and Xueping 2014; Nazeer et al. 2016; Qing et al. 2015; Wu et al. 2017; Yan et al. 2018). The problem with hazardous elements occurs in both high and low income countries (Kanda et al. 2018). Not only are industrial sites affected by HM enrichment. The increase in HMs in sediments of urban water bodies has also been studied in the recent years, showing an accumulation of HMs in sediments (mostly fine-grained fraction) often leading to contamination (Alexakis 2011; Devesa-Rey et al. 2011; Omwene et al. 2018; Sekabira et al. 2010). Total concentrations of heavy metals are generally used to evaluate the contamination status of the environment (Kanda et al. 2018). The research interest in sediment contamination also increases due to the fact that they are an important indicator of environmental alteration under anthropogenic influences (Alexakis and Gamvroula 2014; Guo et al. 2019). Sediments are classified as solid environmental materials; situated between the geosphere, the atmosphere, the biosphere, and the hydrosphere, where they represent a major sink for heavy metals released to the environment as the result of human activities. The changes in environmental conditions in and around retention tanks and streams are recorded in the sediment profiles (Silva et al. 2014; Wang et al. 2019). HMs accumulate in sediments in various forms; some of them are likely to be mobilized and become environmentally toxic in the course of utilization (Wen et al. 2016). Within the exchangeable or carbonates-bond fractions of heavy metals released from sediments, only < 1% is considered safe to the environment, and over 50% of the total amount may pose a high risk and possibly enter the food chain (Nayak 2015).

The measures of site contamination with HMs usually incorporate a variety of pollution or enrichment indices—just to mention a few of them: pollution load index (PLI) (Buat-Menard and Chesselet 1979; Hakanson 1980; Håkanson 1984; Kowalska et al. 2018; Likuku et al. 2013; Weissmannová and Pavlovský 2017), enrichment factor (EF) (Buat-Menard and Chesselet 1979; Hakanson 1980; Håkanson 1984; Kowalska et al. 2018; Likuku et al. 2013; Weissmannová and Pavlovský 2017; Yan et al. 2018), anthropogenic factor (AF) (Hakanson 1980; Kowalska et al. 2018; Likuku et al. 2013; Weissmannová and Pavlovský 2017), and modified contamination degree (mCd) (Abrahim and Parker 2008; Likuku et al. 2013). The assessment of HM distribution in sediments and contamination levels provides the basis for consideration of sediments remediation techniques and evaluation of the potential release of HMs into water and transportation downstream.

Scanning metal concentration in sediments is an effective tool reflecting the contamination existing in the watershed that should be of interest for decision-makers while developing effective environmental strategies to protect the public and health of the ecosystem. Our study investigates the geochemistry of sediments in Gdansk (northern Poland) where increasing development and population growth (from approx. 450,000 to almost 600,000) in the previous two decades took place, which was likely to contribute to environment contamination.

Gdansk is almost 600,000 inhabitants located on the southern coast of the Baltic Sea (Gulf of Gdansk). The storm water collection systems consist of covered drains discharging into several 10–15 km long streams that feed into the Gulf of Gdansk. To minimize flood risk, over 50 retention tanks (RTs) were constructed along the streams. During the flood events, the accumulated sediments can be redistributed inside the stream/retention tanks system or transported out of the catchment, carrying pollutants along with them (Dias-Ferreira et al. 2016). In the case of Gdansk, the final recipient is the Baltic Sea. The last major flood episode in Gdansk, which took place in July 2016, turned out to have a huge impact on the quality of bottom sediments and HMs transport downstream (Nawrot et al. 2018; Wojciechowska et al. 2017). The trapping of HMs in sediments can lead to environmental or health risks which is an issue of the highest significance in densely populated areas. On the other hand, HM deposition counteracts transportation downstream and accumulation in the sea. This is of importance since the Baltic Sea, as the largest semi-closed sea in the world with a densely populated watershed, is particularly vulnerable to all types of contaminants including nutrients, micropollutants, and HMs (Kiedrzyńska et al. 2014).

The main objective of our study was the assessment of HM distribution (spatial and vertical) and enrichment in bottom sediments collected from four RTs along the Strzyza Stream. The HMs typically associated with human activities were studied: zinc (Zn), copper (Cu), lead (Pb), cadmium (Cd), chromium (Cr), and nickel (Ni). Differences between natural and anthropogenic levels of HMs were quantified by calculating the enrichment factor (EF) and anthropogenic factor (AF). Based on the AF, the modified contamination degree (mCd) was calculated to assess the overall average value for a range of HMs analysed. Multivariate statistical analysis (correlation analysis (Spearman Rank), hierarchical cluster analysis (CA), and factor analysis (FA)) were applied to evaluate the results. The sequential extraction of heavy metals was used to assess the mobility of the studied metals in the aquatic environment.

The results of this case study contribute to the assessment of urbanization impact on freshwater bodies and can be helpful in defining quality limits and planning mitigation strategies. In this case, Strzyza Stream can be looked at as an exemplary urban stream, and the outcomes of the study are not only of local interest but also have wider implications. Moreover, there are few studies reflecting the HM pollution associated with urban activities in the Baltic Sea region as an area particularly vulnerable to contamination.

## Materials and methods

### Study area

**Fig. 1:**
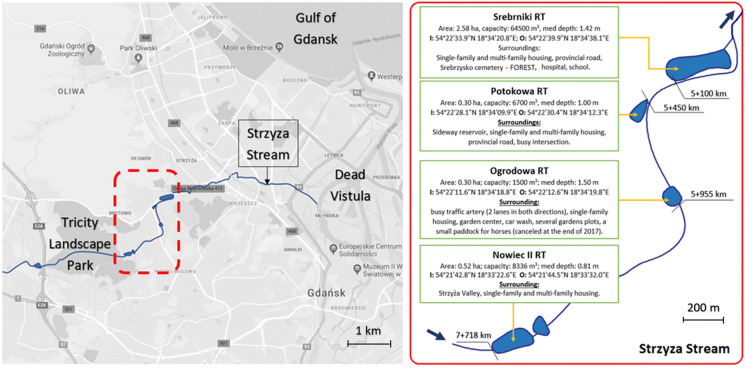
Location of the Strzyza Stream and analysed RTs on the map of Gdansk

The Strzyza Stream is 13.2 km long and represents the longest in Gdansk. The catchment area covers about 34 km^2^. Average flow rates in the middle stream range from 0.076 to 0.087 m^3^/s; in the lower part, the flow rate reaches 0.152 m^3^/s. In the upper part, the stream cuts a deep erosion valley through the Tricity Landscape Park, covered with forest and has quite a high hydraulic slope of 12.2%. Right below the Nowiec II RT (7 km + 718) the stream inflows to the urbanized area. The stream outflows to the Dead Vistula 5 km further before its discharge into the Baltic Sea. The Strzyza Stream is a recipient of the storm water drainage system along its entire length. In practice, it is an interceptor (partly covered and partly open) of storm water from a relatively large semi-urban and urban area. There are 9 RTs along the Strzyza Stream, with a total retention capacity of 215,000 m^3^ and total area of about 13.5 ha. Figure 1 presents the location of four analysed tanks (Nowiec II (7 km + 718), Ogrodowa (5 km + 995), Potokowa (5 km + 450), and Srebrniki (4 km + 730)) located in the middle run of the stream.

### Sampling and measurements

#### Sample collection

The samples of sediments from RTs were collected in the period from June to August 2017 (three series of sampling). The samples were collected from four tanks along the Strzyza Stream: Nowiec II, Ogrodowa, Potokowa and Srebrniki at two points for each tank: near the inflow (I) and outflow (O). At each measurement point, the sediment core was collected from the following layers: 0–2 cm, 8–10 cm, 16–18 cm, 24–26 cm (each of the layers is the result of mixing two samples taken side by side). In total, ninety-six sediment samples were taken with a plastic sampler for core extraction. The samples were placed in clean polyethylene packages and delivered to the laboratory within 4 h from extraction.

#### Measurement of HM concentrations

The collected sediments samples were put in Petri dishes and lyophilized in Labconco Freezone. The next step involved passing sediments through 2.0 mm sieves. Contact between the sediments and metals was avoided throughout the preparation process to exclude external contamination.

Total HM concentration: a sediment subsample of 0.5 g (0.001 g accuracy) was weighted into Teflon bombs. Extra pure chemical reagents—HClO_4_, HF and HCl (3:2:1; Suprapur)—were added. Subsequently, the Teflon test tubes were placed in an oven (140 °C) for 4 h. Then, the solution was evaporated to dryness and 5 ml of concentrated HNO_3_ (Suprapur) was added and evaporated. The dried residue was dissolved in 10 ml of 0.1 M HNO_3_ (Suprapur) and transferred to polypropylene test tubes.

Sequential extraction procedures of HMs: a sediment subsample of 1 g (0.001 g accuracy) was subjected to BCR sequential extraction with diluted acetic acid (0.11 M CH_3_COOH), a reducing agent (hydroxylammonium chloride 0.05 M NH_2_OH·HCl, pH2) and an oxidant (hydrogen peroxide H_2_O_2_ 8.8 mol/l).

Previously prepared solutions were used, with the exception of a NH_2_OH·HCl solution, which was prepared just before use. Immediately after completing stage one, the next extraction phase was started. The extraction of sediment samples to obtain each fraction was carried out at room temperature for 16 h. The obtained solutions were transferred to polyethylene tubes, after which they were acidified with concentrated HNO_3_ acid and stored until analysis.

Dilutions of × 10, × 100 and × 1000 were prepared. The solutions were analysed for Zn, Cu, Pb, Cr and Ni with a flame atomic absorption spectrometer (AAS) and for Cd by inductively coupled plasma mass spectrometry (ICP-MS). The results are presented in mg/kg d.w. The measurements were carried out in three replications. Quality control was assured by analysing certified reference sediments (IAEA-433 and JMS-1 and “blanks”, according to the same procedure). Recoveries in the range of 92–103%, depending on individual metals, were achieved, thus indicating good agreement between certificated and analytical values. The precision, given as Relative Standard Deviation, was in the range of 3–5%. The detection limits (LOD) of each element was calculated as Blank + 3·SD, where SD values were the standard deviations of the blank samples (*n* = 5). LODs were as follows: Zn = 0.5 µg/g, Cu = 0.3 µg/g, Pb = 1.0 µg/g, Cr = 1.5 µg/g, Ni = 0.7 µg/g, Cd = 35 ng/g (0.035 mg/g).

#### Granulometric analysis

To determine the particle size distribution of the sediment samples, a granulometric analysis was performed using six normalizing sieve apertures (< 0.0625 mm, 0.125 mm, 0.250 mm, 0.500 mm, 1.0 mm, and 2.0 mm). 250 g of dried sediments were sieved in 15 min in a mechanical sieve shaker. The residuals on each of the sieves were weighed (0.01 g accuracy). The percentage content of each fraction: mud (< 0.063 mm), sand (0.063–2.0 mm), and gravel (> 2.0 mm) was determined.

### Evaluation of sediments contamination with HM

#### Enrichment factor

The EF coefficient is used to estimate the enrichment of the surface layer of sediments with HMs. EF allows to eliminate the potential impact of differences in grain size sediments of the surface layer and sediments of a geochemical background (Buat-Menard and Chesselet 1979; Hakanson 1980; Håkanson 1984; Kowalska et al. 2018; Likuku et al. 2013; Weissmannová and Pavlovský 2017; Yan et al. 2018). EF was calculated according to formula (1):

1 $$ {\text{EF}} = \frac{{C_{n} }}{{C_{\text{ref}} }} \div \frac{{B_{n} }}{{B_{\text{ref}} }} $$

where $C_{n}$ is the concentration of HM in the environment under study, $C_{\text{ref}}$ is the concentration of HM in the reference environment, $B_{n}$ is the concentration of the reference chemical element in the environment under study and $B_{\text{ref}}$ is the concentration of the reference element in the reference environment. In this paper, Fe was used as a reference element. Fe and HM concentrations in the bottom layer (24–26 cm) of Nowiec II RT sediments were used as reference values $B_{\text{ref}}$ and $C_{\text{ref}}$. Sediments from the Nowiec II RT generally had the lowest contents of HMs; on the level close to the values reported by Polish Geological Institute National Research Institute (GeoLOG) for the surface soil layer in the analysed area (Cu-5.1, Zn-35, Pb-12.4, Cd-0.50, Ni-4.1, Cr-5.1, Fe-7900 [mg/kg d.w.]). The classes of contamination due to EF are shown in Tab.S. 1.

#### Anthropogenic factor

The AF allows to estimate the increase in metal concentrations in the top layer of sediments in relation to deeper layers—background. The AF coefficient is calculated from the formula (2) (Hakanson 1980; Kowalska et al. 2018; Likuku et al. 2013; Weissmannová and Pavlovský 2017):

2 $$ {\text{AF}} = \frac{{C_{n} }}{{C_{\text{ref}} }} $$

where $C_{n}$ is as and $C_{\text{ref}}$ is the concentration of examined HM in the reference environment. For AF calculations, the $C_{\text{ref}}$ was taken for each calculation point from the deepest core layer (24–26 cm) of each RT. The AF > 1 indicates the enrichment of the layer with analysed element with reference to the deeper layer. The factor based on calculation for each element is also known as contamination factor (CF). Sediment classes according to AF are given in Tab.S. 1.

#### Modified degree of contamination

The method developed by Abrahim and Parker (2008) and Likuku et al. (2013) allows for mCd calculations without an upper limit of HM concentration. The calculations were performed according to formula (3):

3 $$ m{\text{Cd}} = \frac{1}{N}\sum\limits_{i = 1}^{N} {{\text{AF}}_{i} } $$

where *N* is the number of elements analysed and AF is the anthropogenic factor.

### Statistical analysis

Correlations of different elements in subsequent core layers of sediments from RTs were calculated using the nonparametric Spearman rank method. The relationships between HMs and between a given metal and percentage share of granulometric fractions (< 0.063 mm, 0.063–2.0 mm) were analysed. Ward’s cluster analysis (CA) and factor analysis (FA) for measured HMs were carried out. In the factor analysis, the number of factors was determined on the basis of the Cattell’s test as 2. The data analysis was performed using STATISTICA software.

## Results and discussion

### Granulometry of sediments

**Fig. 2:**
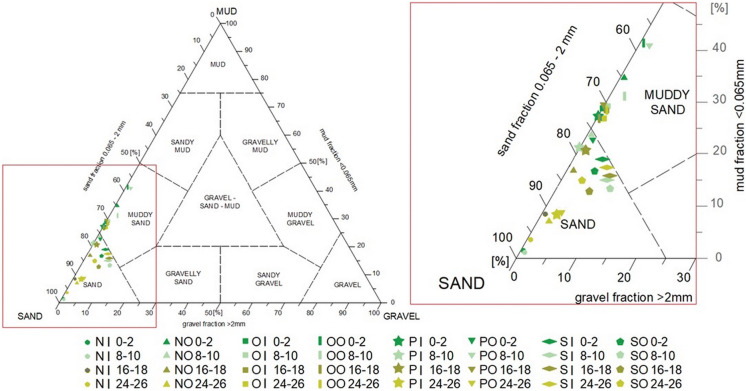
Sediments classification—grain triangle (abbreviations: N—Nowiec II RT, O—Ogrodowa RT, P—Potokowa RT, S—Srebrniki RT, I—inflow, O—outflow)

Sediments deposited in the RTs are mainly composed of muddy sand and sand (58–100% of sand fraction 0.065–2 mm) (Fig. 2). The top layer (0–2 cm) of Ogrodowa RT (O) and the layer 8–10 cm of Potokowa RT (O) had the highest content of fine fraction (< 0.063 mm), about 40% for both sites. In the case of gravel fraction > 2 mm, the layers of sediments from Srebrniki (I and O) contained about 7–10% of this fraction (the highest value). In other sediment samples, the content of the fraction > 2 mm was below 5%. The granulometry results confirmed that with the decrease in flow velocity in the retention tank, the larger and heavier fraction of suspended solids were deposited in the immediate vicinity of to the inlet, while the smaller and lighter fraction settled towards the outlet (Farkas et al. 2007). At the inlet (points I in this study) in the top layer (0–2 cm), the percentage share of fraction > 0.063 mm is higher than the share of fine fraction < 0.063 mm, while in points O (outlet) there is an inverse relationship. It has been widely observed that HMs are predominantly associated with the finer fraction of sediments (Ji et al. 2018; Silveira et al. 2016).

### Spatial and vertical distribution of heavy metals

**Fig. 3:**
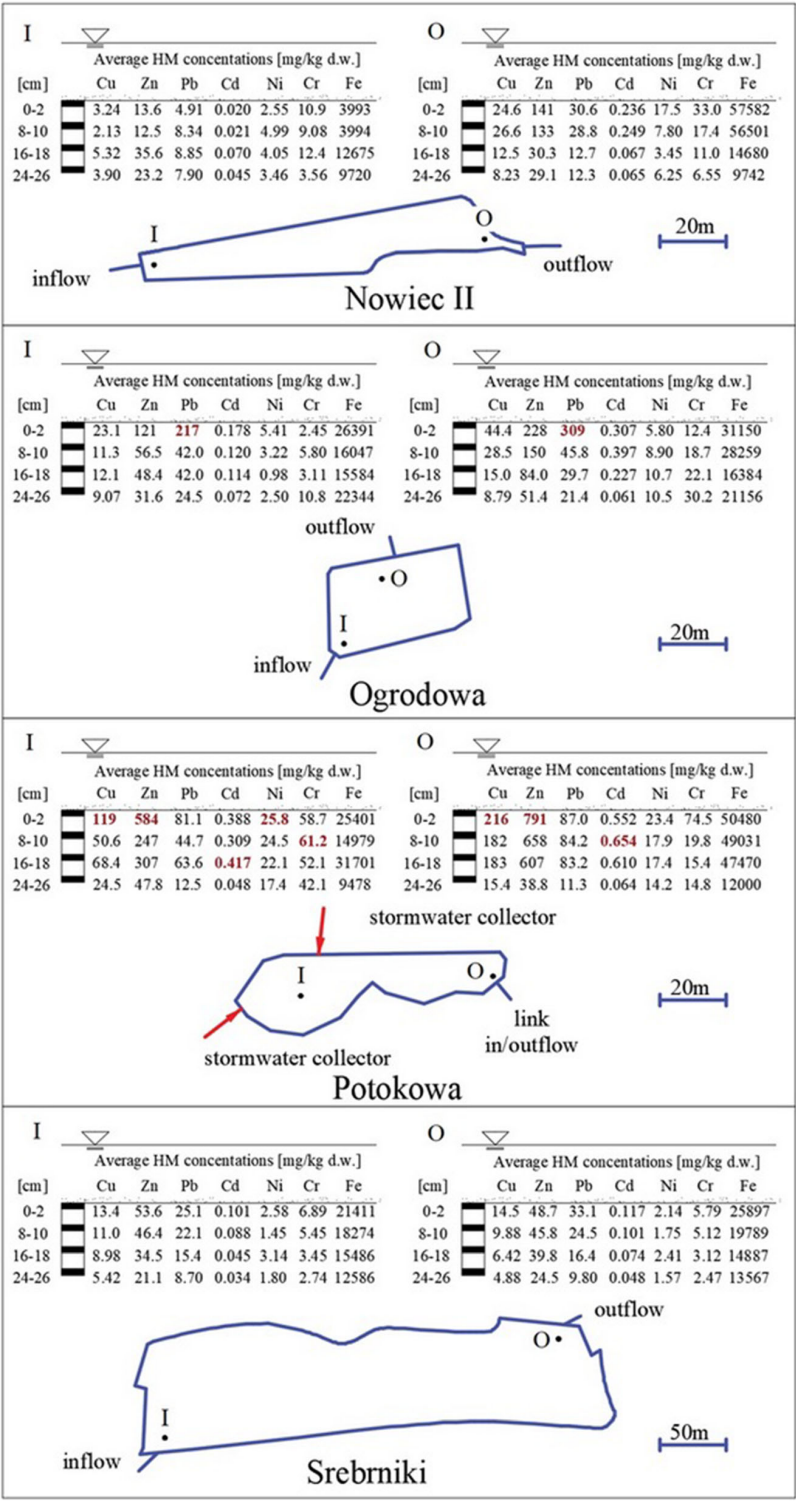
Average HM concentrations (mg/kg d.w.) in vertical profiles of sediments collected from RTs along the Strzyza Stream

The average concentrations of metals in the vertical cores of sediments are presented in Fig. 3 and in Tab.S. 2. The rank order of concentrations of the studied HMs, from highest to lowest, was as follows: Fe, Zn, Cu interchangeably with Pb, Cr, Ni and Cd. Iron concentrations ranged from 3993 mg/kg d.w. for the top layer in I Nowiec II RT to 57582 mg/kg d.w. in O Nowiec II RT (top layer). The low concentration of Fe at I Nowiec II is assumed to be connected to significant flow velocity at the inflow to this RT; thus the leaching of iron compounds from the surface layer of sediments may occur. Elevated Fe concentrations in shallower sediment layers in comparison to the geochemical background in the surface soil layer (7900 mg/kg d.w.) (GeoLOG) may indicate an anthropogenic origin. In all RTs, the enrichment of Zn, Pb, Cu, Cd occurred in the top 18 cm of the sediment cores.

In general, the lowest HM content was recorded in the Nowiec II RT, which is located on the Strzyza Stream just below the “Strzyza Valley” Tricity landscape park, where little or no anthropogenic interference is expected. The nature reserve was established by the regional authorities in 2007 to protect the alluvial forests and plant species. Previously, a military training ground was located there.

The highest concentrations of Pb were observed in the Ogrodowa RT. Regarding the natural geochemical background (GeoLOG), the ratio of Pb in Ogrodowa (O) is up 30 times higher. In the top layer of the Potokowa RT, the highest concentrations of Cu and Zn were observed. According to WHO (2001) the natural background level of Zn is usually found up to 100 mg/kg d.w., which means that the maximum obtained value at Potokowa (O) exceeds 8 times this value. In addition, in the Potokowa RT the highest concentrations of Ni, Cr, and Cd were observed. The maximum Cd concentrations occurred in the deeper layers (16–18 cm (I) and 8–10 cm (O)). The distribution of HMs in the vertical profile manifested an increase in the content of Cu, Zn, Pb, and Cd with decrease in depth. No obvious tendency for Cr and Ni was observed. The Ogrodowa and Potokowa RTs are located close to each other and near main street traffic. Additionally two outlets of the storm water drainage system discharge into the Potokowa RT. Nearby, there are allotment gardens and a wholesaler of ornamental pot plants. These aspects might explain the increased concentrations of Pb, Cu, Zn, Cd (Fig. 3), which are most likely associated with external influence. Unusually higher concentration of HMs in the deeper layer of the sediments suggests a “historical” deposition of the investigated metals in the area. Sediments are dredged from the retention tanks once every 4–7 years. The concentrations of Cd in the top layer in the I and O Potokowa RT indicate that accumulation of Cd in “freshly” deposited sediments is smaller than previously.

In comparison to river sediment studies carried out in an urban catchment area in England (Hurley et al. 2017), the concentration ranges for Cu (53.1–383 mg/kg d.w.) and Pb (80.4–442 mg/kg d.w.) were similar. However, the Cr and Zn concentrations reported by Hurley et al. (2017) were definitely higher than in case of the Strzyza RTs and Cr ranged from 76.5 to 413 mg/kg d.w. (with a minimum value of a similar level as the maximum value obtained in this study) and for Zn from 282 to 1020 mg/kg d.w.. The maximum concentrations of Zn (1466 mg/kg d.w.), Pb (788 mg/kg d.w.) and Cd (19.5 mg/kg d.w.) reported for bottom sediments in an urban catchment in Portugal—the Mondego River (Dias-Ferreira et al. 2016)—were significantly higher than those obtained in our study. On the other hand, the concentrations of Ni (43 mg/kg d.w.) and Cr (63 mg/kg d.w.) measured in sediments from the Mondego River were similar to the concentrations in the RTs along the Strzyza Stream, while the maximum Cu concentration for the Mondego River (79 mg/kg d.w.) was almost 3 times lower than the maximum concentration measured in this study (for the top layer of sediments from the Potokowa RT).

In comparison to the former study carried out in the Strzyza RTs in spring period (March–May 2017) (Wojciechowska et al. 2019), the concentrations of HMs in the summer period increased for all the analysed HMs in the Nowiec II and Potokowa RTs. Furthermore, in Ogrodowa Cu, Zn and Pb concentrations increased in the summer season, while Cr and Cd concentrations decreased. The observed changes may result from the increase in air and water temperature in the summer season leading to lower dissolved oxygen concentrations in the water column, especially near the bottom. Additionally, the content of organic matter is higher in the summer period. Thus, when HMs mix with organic and residual fraction (stable), the HM concentration will be higher at the end of the summer (when more organic matter has been accumulated). Cd is often associated with the mobile fractions and more easily released into the water column, which may explain its lower concentration in the summer period.

To support these considerations the preliminary results of speciation analyses (Tab.S. 3) based on the top layer of the sediments collected during this investigation proved that Zn, Cu, Pb, Ni, and Cr were connected with stable fractions (residual and organic). In general, Cd was the only metal bound to fractions with high mobility and readily available to the biome. The share of Cd in different fractions is as follows: most Cd was related to the fraction of iron-magnesium oxides alternating with the exchangeable fraction > Cd related to the fraction of organic matter > Cd linked with the residual fraction. The first two fractions can undergo chemical changes at the sediment–water interface and are susceptible to remobilization in water (Lundy et al. 2017). Water pH and elevated chloride concentrations tend to enhance chloride complex formation, which decreases the adsorption of Cd in the sediments, thereby increasing Cd mobility (Islam et al. 2015). Our findings confirm high Cd mobility, which is highly dangerous to the biome due to its easy assimilation and high capability of accumulation in plant and animal tissues. Other investigated HMs were mostly bound to fractions 3 and 4, which are considered as immobile and not participating in the chemical reactions in spite of environmental changes (Peng et al. 2009). Only in the case of Zn and Cu in the Nowiec II RT was their relation to the second fraction observed, which is associated with iron and manganese oxides.

### Metal enrichment evaluation basing on pollutants indices

#### Heavy metal enrichment factor

**Fig. 4:**
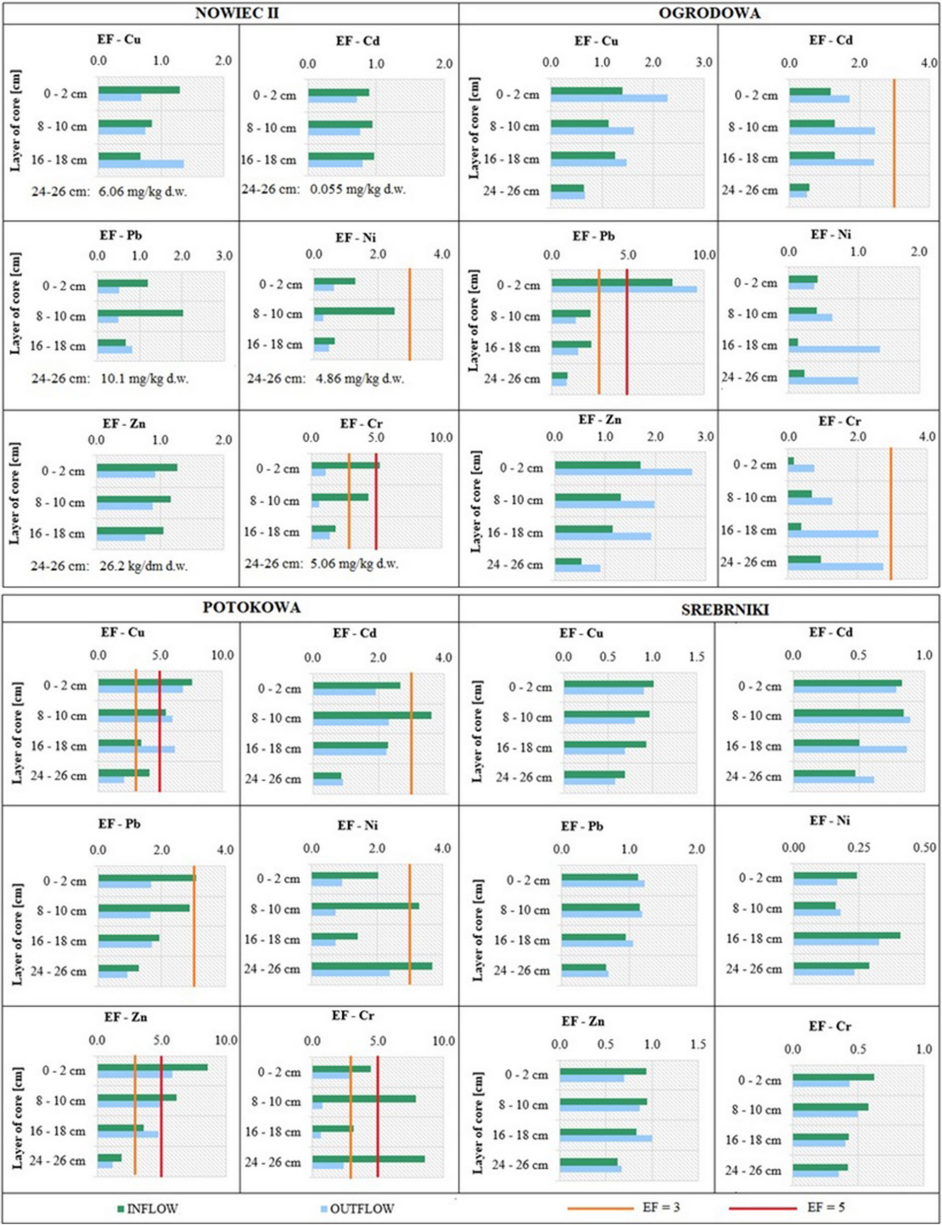
Enrichment factor in the vertical profiles of sediments deposited in RTs along the Strzyza Stream

EF is a useful tool for distinguishing between anthropogenic and natural sources for elemental enrichment (Alahabadi and Malvandi 2018). The greater the EF, the higher the level of sediments contamination with HMs (Wang et al. 2012). However, the main question is selection of appropriate geochemical background concentrations for EF calculation. Different approaches were adapted in the previous studies: concentration of HM in the deepest layer of core profiles (Birch and Olmos 2008); or concentrations measured upstream where no pollution is expected (Grosbois et al. 2006). In the recent years, there was no research focused on the sediments cores from RTs in Gdansk, thus the quantification of HMs enrichment (Fig. 4) is referred to the layer 24–26 cm from Nowiec II RT due to minor probability of anthropogenic interference. EF values in the range from 0.05 to 1.5 represent natural origins of HMs and EF higher than 1.5 suggests anthropogenic origin (Chen et al. 2015; Malvandi 2017). In addition, the EF values < 1 indicate a depletion of the element and reflect the crustal source of the elements in sediments (Hanif et al. 2016).

Generally, low EF values were found for sediments deposited in the Nowiec II RT. No enrichment of Cd in any of the analysed core samples was noted. The EF = 1 value was slightly exceeded for Zn, Cu, Ni, and Pb, with the maximum of EF_Pb_ = 2.0 and EF_Ni_ = 2.3 in the 8–10 cm sub-layer. The exception from the decidedly low EF values were the results obtained for EF_Cr_. In the top layer at the site I in Nowiec II RT, the value of EF_Cr_ = 5 was observed; the EF_Cr_ values decreased with depth. The increase of EF in subsequent RTs (from Nowiec II to Potokowa) was noted, which was in agreement with increasing urbanization of the Strzyza watershed. This growing accumulation trend was disturbed by Srebrniki RT, where low EF values (< 1.5) were found. It could be stated that the Potokowa RT located at a short distance upstream of the Srebrniki RT acted as a buffer zone and retained most of the contamination associated with surface runoff and suspended solids. Furthermore, no direct inlets from the storm water drainage system discharge into the Srebrniki RT; in addition the retention tank is surrounded by vegetation buffer zones (min 3 m wide) which prevent direct runoff from paved areas. Moreover, the cemetery area located close to this RT is in fact a forest area.

According to Tab.S. 1, the EF results show that sediments in the Ogrodowa and Potokowa RTs are moderately enriched with HMs originating from anthropogenic sources. Traffic and atmospheric deposition are most likely the most significant sources of HMs. EF values for Cu, Zn and Cd in these RTs hovered in the range of 1–2.5 with a maximum EF_Zn_ = 2.7 in the top layer. Moderately severe enrichment was noted for Pb in the top layers, which was most likely related to traffic. EF_Pb_ in the top layer was higher in Ogrodowa (EF_Pb_ = 8, EF_Pb_ = 9.8 at I and O) than in Potokowa (EF_Pb_ = 4, EF_Pb_ = 1.8 at I and O). The Pb deposition in both RTs seemed quite fresh, because a high EF value occurred only in the top layer in Ogrodowa and the two top layers in Potokowa. EF_Cd_ hovered around the 2 mark in both RTs, and however, moderate enrichment (EF_Cd_ = 3.8) occurred in Potokowa I 8–10 cm. The elevated values of the EF_Cd_ are alarming since Cd has a high geochemical activity in the environment and may be transported in the river system for long distances as Cd has a higher mobility and water solubility than other HMs (Ghrefat et al. 2011).

#### Metal anthropogenic factor

The enrichment of sediment samples from different core layers for a given HM was also assessed according to the AF (Fig.S. 1). The AF reflects the HM enrichment of subsequent layers in relation to the bottom layer (24–26 cm) of each core. The AF values for Nowiec II showed low contamination at site I for Cu, Zn, Pb, Cd, and Ni for subsequent layers of the core, while at site O the AF values hovered around the 2 mark (moderate contamination) for AF_Pb_ and close to 3 (moderate/considerable contamination) for AF_Cu_ and AF_Zn_. AF_Cr_ at I was around 3 for all layers; considerable level of contamination—AF_Cr_ = 5 was noted at O (0–2 cm).

Moderate AF values in Srebrniki RT were obtained. The maximum values for 0–2 cm layer for AF_Cu_ = 3 (O), AF_Pb_ = 3.4 (O) and AF_Cd_ = 3 (I) were noted. The distribution of HMs in this RT increased with the depth.

The AF values for the Ogrodowa RT at 8–10 cm and 16–18 cm layers were below 3, with the exception of AF_Cd_ values at O (all layers)—which suggests considerable contamination with Cd. The top layer of sediments was highly contaminated with Pb at I and O (AF_Pb_ ≥ 6) which is consistent with the CF values (Fig. 4). The top layer of sediments was also considerably contaminated with Zn (I and O) and at O with Cd and Cu.

In the Potokowa RT, the values of AF ≥ 6 were observed in subsequent layers of core for Cd (I and O), Cu (O), Zn (O) and Pb (O). Considerable pollution was noted for Cu (I) and Cr (O) in the top layer, Zn and Pb (at I) in 8–10 cm and 16–18 cm layers. The AF for Ni and Cr (except AF_Cr_ at O 0–2 cm layer) hovered around the 2 mark.

The AF analysis results indicate that the sediments from two tanks—Ogrodowa (at O) and Potokowa (O)—were constantly supplemented with HMs: Ogrodowa with Cd, and Potokowa with Cu, Zn, Pb (at O) and Cd (at I and O), although the storm water drainage system is equipped with sedimentation tanks and separatory funnels. The results of index calculations and concentrations of HMs show that these safeguards may not be enough to prevent HM flux. Different solutions are needed to mitigate HM pollution. In this context, the vegetation buffer zones (even greenery or grass “strips”)—similar to those around the Srebrniki RT as well as other Green Infrastructure solutions—may seem fairly efficient.

#### Metal modified degree of contamination

The mCd values calculated based on the AF are presented in Table 1. The gradation proposed for mCd by Abrahim and Parker (2008) is as follows: mCd < 1 mean to a very low degree of contamination, 1.5 ≤ mCd < 2*—*low degree of contamination, 2 ≤ mCd < 4*—*moderate degree of contamination, 4 ≤ mCd < 8*—*high degree of contamination, 8 ≤ mCd < 16*—*very high degree of contamination, 16 ≤ mCd < 32*—*extremely high degree of contamination, mCd ≥ 32*—*ultra high degree of contamination.

**Table 1:** Modified degree of contamination in the vertical profile of sediments deposited in the RTs along the Strzyża Stream (I—inflow, O—outflow)

mCd								
Layer	Nowiec II		Ogrodowa		Potokowa		Srebrniki	
	I	O	I	O	I	O	I	O
0–2 cm	1.1	3.6	3.4	5.0	5.8	9.6	2.5	2.4
8–10 cm	1.1	3.0	1.4	2.7	3.4	8.2	2.0	1.9
16–18 cm	1.7	1.1	1.1	1.7	4.2	7.8	1.6	1.5
24–26 cm	1.0	1.0	1.0	1.0	1.0	1.0	1.0	1.0

In relation to the deepest layer of the core, a moderate degree of contamination was observed at O (0–2 cm, 8–10 cm) in the Nowiec II RT. A low degree of contamination for 16–18 cm layers (I and O) was noted in the Ogrodowa tank, while in the top layers moderate (at I) and high (at O) degrees of contamination were observed. The highest mCd values were observed at I and O in the Potokowa RT, where the value of mCd for the top layer at O suggests a very high degree of contamination. In the case of the Srebrniki RT, only low contamination was observed.

### Correlation analyses

Complex inter-relationships between different HMs in sediments are usually observed. Numerous factors affect their relative abundance: original contents of HMs in rocks and parent materials, various processes of soils and sediment formation, as well as anthropogenic factors such as contamination by human activities (Sun et al. 2010). In this study, correlation analysis was performed between all analysed metals for the top (0–2 cm), middle (8–10 cm and 16–18 cm) and the deepest core layers (24–26 cm). The correlations in the middle layers were calculated using all measured HM concentrations for both 8–10 cm and 16–18 cm layers. In Table 2 the correlation coefficient matrix is depicted, listing the Spearman’s product moment correlation coefficient.

**Table 2:** Correlation analysis (Spearman’s rho matrix) between analysed HM concentrations in sediment profiles calculated for all samples collected from the RTs along the Strzyza Stream

	Cu	Zn	Pb	Cd	Ni	Cr	Fe
*Top 0–2* *cm*							
Cu	1.00						
Zn	**0.98**	1.00					
Pb	0.01	0.05	1.00	0.25			
Cd	**0.93**	**0.95**	0.25	1.00			
Ni	**0.79**	**0.86**	− 0.18	**0.82**	1.00		
Cr	**0.92**	**0.93**	− 0.24	**0.86**	**0.94**	1.00	
Fe	0.47	0.46	0.06	**0.65**	**0.61**	0.54	1.00
*Middle (8–10 and 16–18* *cm)*							
Cu	1.00						
Zn	**0.99**	1.00					
Pb	**0.89**	**0.91**	1.00				
Cd	**0.92**	**0.95**	**0.94**	1.00			
Ni	**0.68**	**0.76**	**0.72**	**0.79**	1.00		
Cr	0.30	0.40	0.42	0.49	**0.89**	1.00	
Fe	**0.72**	**0.73**	**0.69**	**0.77**	0.46	0.19	1.00
*Bottom 24–26* *cm*							
Cu	1.00						
Zn	**0.71**	1.00					
Pb	0.11	0.52	1.00				
Cd	0.15	0.38	**0.71**	1.00			
Ni	**0.90**	**0.84**	0.07	0.20	1.00		
Cr	**0.83**	**0.93**	0.37	0.14	**0.85**	1.00	
Fe	− 0.21	0.30	**0.89**	0.49	− 0.21	0.11	1.00

Bold—the correlation coefficients are significant with *P* < 0.05; bold—the significant correlations occurring in all analysed layers of core

Correlations between metals in all sediment core layer samples were significant in four cases: between Zn–Cu (*r*_0–2 cm_ = 0.98, *r*_middle_ = 0.99, *r*_24–26 cm_ = 0.71), Ni–Cu (*r*_0–2 cm_ = 0.79, *r*_middle_ = 0.68, *r*_24–26 cm_ = 0.90), Zn–Ni (*r*_0–2 cm_ = 0.86, *r*_middle_ = 0.76, *r*_24–26 cm_ = 0.84), and Cr–Ni (*r*_0–2 cm_ = 0.94, *r*_middle_ = 0.89, *r*_24–26cm_ = 0.85). These high correlations indicate that those pairs of HMs may have a similar contamination level and similar contamination sources (Li et al. 2009). In addition, in each layer of the core, different correlations between metals were found: in the top layer significant correlations occurred between Cd–Cu, Cr–Cu, Cd–Zn, Cr–Zn, Ni–Cd, Cr–Cd, Fe–Cd, and Fe–Ni. High correlation coefficients *r* = 0.84 between Pb–Zn and *r* = 0.64 between Pb–Cu were reported by Hurley et al. (2017) in the Mersey and Irwell catchments in England. In this study, correlations between Pb–Cu and Pb–Zn only occurred in the middle layer (Pb–Cu *r*_middle_ = 0.98 and Pb–Zn *r*_middle_ = 0.95). In the middle and bottom layers, significant correlation occurred between Fe–Pb. Furthermore, strong positive correlations between Fe and Cu (*r*_middle_ = 0.72), Zn (*r*_middle_ = 0.73), Pb (*r*_middle_ = 0.69), Cd (*r*_middle_ = 0.77) were observed in the middle layer. Such positive strong correlations were also reported by Sun et al. (2018), while Li et al. (2001) confirmed that Cu, Zn and Pb and are mainly associated with the residual fraction of sediments. Similar correlations as those in the study by Li et al. (2001) were also observed in our research (Table 3).

Statistically relevant relationships occurred between the percentage share of fine fraction < 0.063 mm and Cu, Zn, Pb, and Cd (Table 3). Numerous studies have confirmed this relationship—together with the increase in the content of fraction > 0.063 mm, the total content of HMs in sediments decreases (Devesa-Rey et al. 2011; Wang et al. 2012). In this study, no correlations were found between Ni and Cr concentrations and the fraction size, in contrast to the research conducted by Ranasinghe et al. (2002) who confirmed that Ni was associated with < 0.063 mm and Cr with < 2 mm fraction. Surprisingly, in our study, the concentrations of these two HMs decreased together with the increase in the content of 0.063–2.0 mm fractions. No statistically relevant correlations between the percentage share of granulometric fraction and Fe were observed.

**Table 3:** Spearman’s correlation matrix between the seven HMs and percentage of granulometric fraction from all samples of sediments collected from the analysed RTs along the Strzyza Stream

	Cu	Zn	Pb	Cd	Ni	Cr	Fe
< 0.063	**0.46**	**0.54**	**0.54**	**0.45**	0.22	0.09	0.36
0.063–2.0	− **0.42**	− **0.49**	− **0.49**	− **0.43**	− 0.15	− 0.05	− 0.34

Bold—the correlation coefficients determined are significant with *P* < 0.05

**Fig. 5:**
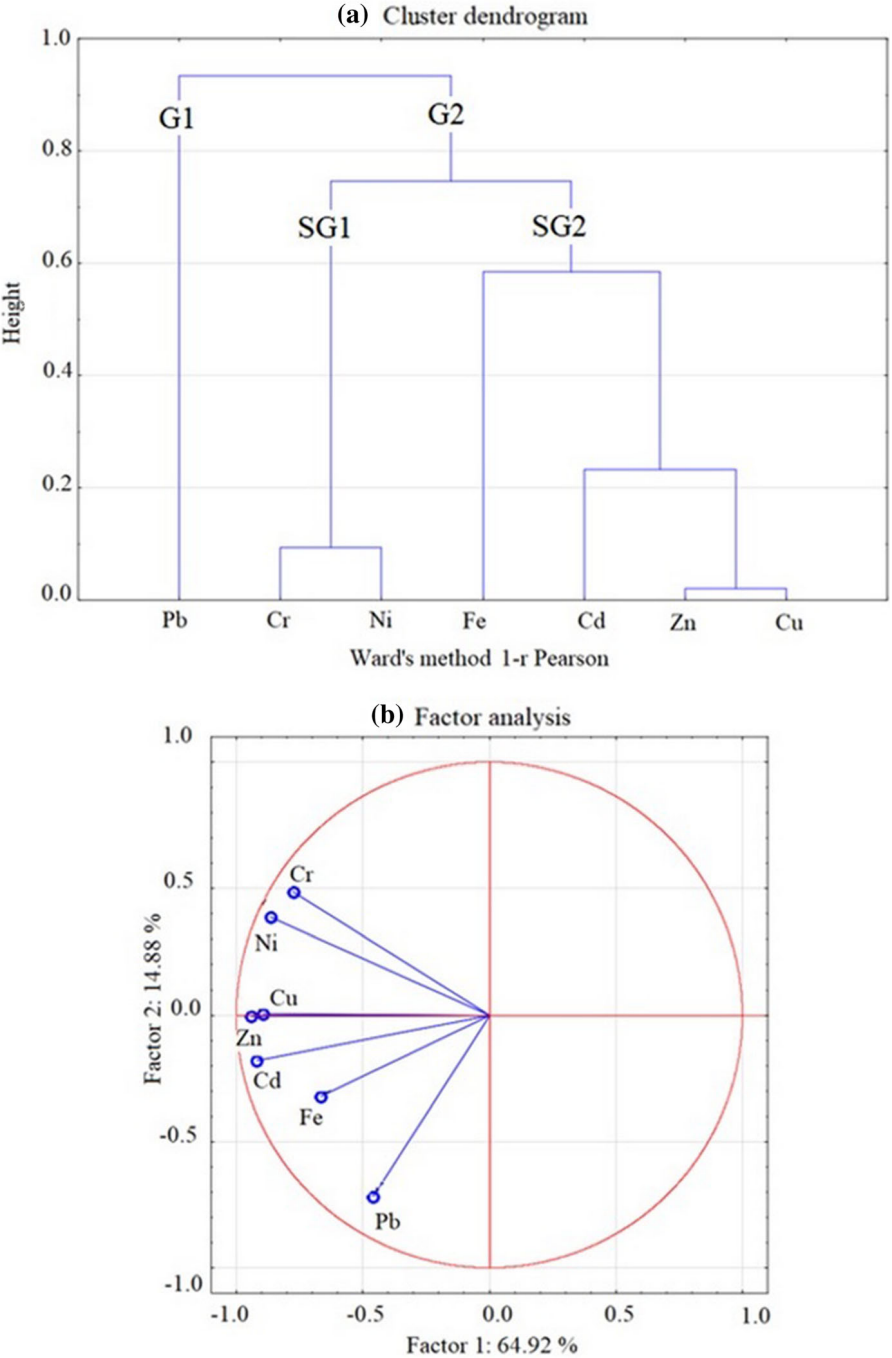
Cluster analysis (**a**) and factor analysis (**b**) of HM concentration in bottom sediments of retention tanks along the Strzyza Stream

Cluster analysis (Fig. 5a) confirmed the mutual significantly correlated spatial distribution between Zn–Cu and Cr–Ni. The Zn and Cu occurrence is strongly connected with the presence of Cd. Cluster analysis distinguished two groups of HMs in the analysed sediments. The first group (G1) contained Pb only, which seems to be due to its highest content in the sediments in comparison to other HMs. The fact that Pb is in a separate group also suggests its different origin. Pb was used as an additive in petrol from the 1990s till the beginning of 2000, when leaded petrol use was banned in EU member states. Since that time, Pb has still been added, although the limit concentration is 5 mg/L due to national requirements. The second group (G2) is divided into two subgroups (SG): SG1 containing: Cr–Ni and SG2 containing: Fe, Cd and Zn–Cu. Factor analysis (Fig. 5b) confirmed that Cr and Ni could originate from other sources than the rest of the metals. Pb, Zn, Cu, Cd, and Pb are the most common HMs emitted by road traffic. According to US EPA (2007) and Dias-Ferreira et al. (2016) at least 90% of Pb, Zn and Cu of the total emission come from vehicle traffic. Hjortenkrans et al. (2006, 2008) distinguished the footprint of different traffic-related HMs in roadside soils. Strong correlations between Cd–Pb and Cu–Zn were found to be a result of braking, that is the wearing of the tyres and cables while braking. A different correlation between Ni–Cr–Cu–Zn was found in road dust by Trujillo-González et al. (2016). These authors state that road impact depends on the type of road considered, with metal contents in road dust being higher in commercial areas than on highways and higher on highways than in residential roads. If adopting assumptions based on the study of Trujillo-González et al. (2016), the highest concentrations of Zn, Pb, Cu and Cd in sediments deposited in the Ogrodowa and Potokowa RTs can be explained since these two retention tanks are located close to a busy traffic artery.

## Conclusions

HMs transported along with surface runoff from urban catchments are deposited in sediments of urban water bodies and in retention tanks. The sequential extraction results confirm that HMs mostly bond to immobile organic and residual fractions. The exception is Cd, with high shares in ion exchangeable and hydroxide fractions, indicating its potential mobility and risk of assimilation by the biome. The highest levels of HM concentrations were found in the Ogrodowa and Potokowa RTs, characterized by intense catchment development and urbanization, suggesting a link between human activities and HM content in sediments. The comprehensive assessment of HM contamination status by EF and AF confirmed that sites Ogrodowa and Potokowa had relatively high sediment pollution levels, with the most Cu, Zn, Cd, and Pb in the top layers of sediments, suggesting systematic enrichment with hazardous elements from surrounding areas. The overall contamination status was assessed with mCd, which recognized the sediment contamination in Srebrniki as low, in Nowiec II as moderate, in Ogrodowa as moderate to high, and in Potokowa as high. The correlation matrixes confirmed similar potential sources for metal pairs: Zn–Cu, Cr–Ni, Zn–Ni, and Cu–Ni. The CA showed that Pb dominated others HMs, and probably originated from a different source. The observation of elevated contents of Pb, Cu, and Zn in all examined sites, in combination with the lack of known industrial source of contamination, indicates the diffuse sources of contamination in the area.

To mitigate HM contamination, the vegetation buffer zones as well as other Green Infrastructure solutions may seem quite effective. The levels of HMs should be monitored prior to the development of utilization strategies for the dredged sediments. Any future research should focus on assessing the isotope of selective metals and selective microbial groups with established knowledge on affinity to specific niches to identify and strengthen the assessment of HM sources. In combination with statistical methods performed in this study, this would help to identify the sources of HM yield and clarify the potentially toxic effects to the biome posed by different levels of HM enrichment in the urban watershed.

## Electronic supplementary material

Below is the link to the electronic supplementary material.

Supplementary material 1 (PDF 326 kb)
