# Supplementary material for: Spatial and vertical distribution analysis of heavy metals in urban retention tanks sediments: a case study of Strzyza Stream
Source: Environ Geochem Health. 2019 Oct 9;42(5):1469–85. doi: 10.1007/s10653-019-00439-8 (PMC7261270; doi:10.1007/s10653-019-00439-8)
Supplement: Supplementary file 1 — Supplementary material 1 (PDF 326 kb) [file 10653_2019_439_MOESM1_ESM.pdf]

Table Suplement 1 Enrichment Factor (EF) and Anthropogenic Factor (AF) description of contamination level (Hakanson, 1980; Wang et al., 2012, Hurley et al., 2017)

EF value	Level of contamination	AF value	Level of contamination
EF<1	No enrichment	AF<1	Low contamination
1≤EF<3	Minor enrichment	1≤AF<3	Moderate
3≤EF<5	Moderate enrichment	3≤AF<6	Considerable
5≤EF<10	Moderately severe enrichment	AF≥6	Very high
10≤EF<25	Severe enrichment		
25≤EF<50	Very severe enrichment		
EF≥50	Ultra-high enrichment		

Table Suplement 2 Average HMs concentrations [mg/kg d.w.] in samples of core sediments in analysed RTs on Strzyza Stream.

Retention tank	Layer of core [cm]	Average heavy metal concentrations in core of bottom sediments [mg/kg d.w.]													
		Cu		Zn		Pb		Cd		Ni		Cr		Fe	
		Inflow I	Outflow O	Inflow I	Outflow O	Inflow I	Outflow O	Inflow I	Outflow O	Inflow I	Outflow O	Inflow I	Outflow O	Inflow I	Outflow O
Nowiec II	0 - 2	3.24 ± 0.03	24.6 ± 0.0	13.6 ± 0.1	141 ± 1	4.91 ± 0.11	30.6 ± 0.3	0.020 ± 0.008	0.236 ± 0.008	2.55 ± 0.08	17.5 ± 0.1	10.9 ± 0.8	33.0 ± 0.7	3993 ± 2	57582 ± 21
	8 - 10	2.13 ± 0.01	26.6 ± 0.1	12.5 ± 0.2	133 ± 2	8.34 ± 0.42	28.8 ± 0.4	0.021 ± 0.003	0.249 ± 0.004	4.99 ± 0.09	7.80 ± 0.06	9.08 ± 0.05	17.4 ± 0.8	3994 ± 3	56501 ± 15
	16 - 18	5.32 ± 0.05	12.5 ± 0.0	35.6 ± 0.3	30.3 ± 0.7	8.85 ± 0.21	12.7 ± 0.2	0.070 ± 0.007	0.067 ± 0.005	4.05 ± 0.07	3.45 ± 0.05	12.4 ± 0.7	11.0 ± 0.5	12675 ± 7	14680 ± 7
	24 - 26	3.90 ± 0.01	8.23 ± 0.10	23.2 ± 0.1	29.1 ± 0.2	7.90 ± 0.26	12.3 ± 0.1	0.045 ± 0.010	0.065 ± 0.007	3.46 ± 0.05	6.25 ± 0.08	3.56 ± 0.04	6.55 ± 0.06	9720 ± 6	9742 ± 4
Ogrodowa	0 - 2	23.1 ± 0.2	44.4 ± 0.2	121 ± 2	228 ± 1	**217 ± 3**	**309 ± 4**	0.178 ± 0.011	0.307 ± 0.001	5.41 ± 0.04	5.80 ± 0.02	2.45 ± 0.05	12.4 ± 0.7	26391 ± 1	31150 ± 7
	8 - 10	11.3 ± 0.1	28.5 ± 0.2	56.5 ± 1.2	150 ± 2	42.0 ± 0.8	45.8 ± 0.7	0.120 ± 0.008	0.397 ± 0.004	3.22 ± 0.08	8.90 ± 0.02	5.80 ± 0.04	18.7 ± 1.0	16047 ± 3	28259 ± 8
	16 - 18	12.1 ± 0.1	15.0 ± 0.1	48.4 ± 0.7	84.0 ± 0.8	42.0 ± 0.7	29.7 ± 0.5	0.114 ± 0.002	0.227 ± 0.003	0.98 ± 0.11	10.7 ± 0.4	3.11 ± 0.03	22.1 ± 0.7	15584 ± 6	16384 ± 9
	24 - 26	9.07 ± 0.15	8.79 ± 0.09	31.6 ± 0.5	51.4 ± 1.4	24.5 ± 0.5	21.4 ± 0.7	0.072 ± 0.001	0.061 ± 0.001	2.50 ± 0.06	10.5 ± 0.3	10.8 ± 0.7	30.2 ± 1.3	22344 ± 4	21156 ± 11
Potokowa	0 - 2	**119 ± 2**	**216 ± 2**	**584 ± 4**	**791 ± 9**	81.1 ± 1.4	87.0 ± 2.0	0.388 ± 0.001	0.552 ± 0.003	**25.8 ± 0.4**	**23.4 ± 0.4**	58.7 ± 1.1	**74.5 ± 1.7**	25401 ± 3	50480 ± 12
	8 - 10	50.6 ± 0.5	182 ± 1	247 ± 1	658 ± 4	44.7 ± 0.7	84.2 ± 0.8	0.309 ± 0.003	**0.654 ± 0.007**	24.5 ± 0.5	17.9 ± 0.3	**61.2 ± 0.9**	19.8 ± 0.8	14979 ± 2	49031 ± 14
	16 - 18	68.4 ± 0.7	183 ± 2	307 ± 2	607 ± 2	63.6 ± 1.2	83.2 ± 1.2	**0.417 ± 0.002**	0.610 ± 0.004	22.1 ± 0.8	17.4 ± 0.1	52.1 ± 0.8	15.4 ± 0.5	31701 ± 8	47470 ± 8
	24 - 26	24.5 ± 0.8	15.4 ± 0.7	47.8 ± 1.6	38.8 ± 0.4	12.5 ± 0.4	11.3 ± 0.1	0.048 ± 0.004	0.064 ± 0.003	17.4 ± 0.2	14.2 ± 0.2	42.1 ± 0.8	14.8 ± 0.4	9478 ± 3	12000 ± 5
Srebrniki	0 - 2	13.4 ± 0.3	14.5 ± 0.9	53.6 ± 0.9	48.7 ± 0.8	25.1 ± 0.8	33.1 ± 0.3	0.101 ± 0.003	0.117 ± 0.004	2.58 ± 0.02	2.14 ± 0.01	6.89 ± 0.05	5.79 ± 0.06	21411 ± 5	25897 ± 7
	8 - 10	11.0 ± 0.7	9.88 ± 0.08	46.4 ± 0.7	45.8 ± 1.1	22.1 ± 1.1	24.5 ± 0.5	0.088 ± 0.003	0.101 ± 0.007	1.45 ± 0.03	1.75 ± 0.03	5.45 ± 0.02	5.12 ± 0.03	18274 ± 3	19789 ± 6
	16 - 18	8.98 ± 0.11	6.42 ± 0.14	34.5 ± 0.5	39.8 ± 0.8	15.4 ± 0.6	16.4 ± 0.1	0.045 ± 0.011	0.074 ± 0.012	3.14 ± 0.04	2.41 ± 0.01	3.45 ± 0.02	3.12 ± 0.01	15486 ± 3	14887 ± 3
	24 - 26	5.42 ± 0.13	4.88 ± 0.07	21.1 ± 0.4	24.5 ± 0.4	8.70 ± 0.23	9.80 ± 0.14	0.034 ± 0.008	0.048 ± 0.002	1.80 ± 0.01	1.57 ± 0.04	2.74 ± 0.03	2.47 ± 0.02	12586 ± 5	13567 ± 3

Bold - the highest HM concentrations obtained

Table Suplement 3 Results of sequential extraction analysis performed for top layer of bottom sediments in Nowiec II and Potokowa RTs on Strzyza Stream.

Retention tank	Measurement point	Fractions of sequential extraction analysis									Percentage of each fraction in the analyzed sample [%]			
		I	SD	II	SD	III	SD	IV	SD	Total of I-IV	I	II	III	IV
		[mg/kg d.w.]												
						Zn								
Nowiec II	I	0.862	0.026	0.278	0.008	0.478	0.016	11.3	0.3	12.9	8	3	6	83
	O	0.867	0.026	29.4	0.847	55.6	1.8	50.9	1.4	137	1	22	41	36
Potokowa	I	6.04	0.18	28.0	0.806	320	11	224	6	578	1	6	55	38
	O	6.20	0.19	31.0	0.892	401	13	337	9	775	1	4	52	43
						Cu								
Nowiec II	I	<0.05	-	1.40	0.042	1.42	0.071	0.367	0.020	3.19	0	44	45	11
	O	<0.05	-	<0.05	-	<0.05	-	24.6	1.4	24.6	0	0	0	100
Potokowa	I	<0.05	-	<0.05	-	<0.05	-	118	6	118	<1	<1	<1	99
	O	<0.05	-	<0.05	-	52.1	2.6	162	9	214	<1	<1	24	75
						Pb								
Nowiec II	I	<0.15	-	<0.15	-	0.198	0.010	4.69	0.21	4.9	0	0	4	96
	O	<0.15	-	<0.15	-	9.15	0.46	21.2	0.9	30.4	<1	<1	30	69
Potokowa	I	<0.15	-	<0.15	-	8.88	0.44	70.4	3.1	79.2	<1	<1	11	87
	O	<0.15	-	<0.15	-	6.58	0.33	79.9	3.5	86.5	0	0	8	92
						Ni								
Nowiec II	I	<0.7	-	<0.7	-	<0.7	-	2.45	0.08	2.45	<1	<1	<1	97
	O	<0.7	-	<0.7	-	4.36	0.19	12.8	0.4	17.2	<1	<1	25	73
Potokowa	I	<0.7	-	<0.7	-	3.41	0.15	21.1	0.7	24.5	<1	<1	15	83
	O	0.991	0.050	<0.7	-	3.36	0.15	18.5	0.6	22.9	<1	<1	17	81
						Cr								
Nowiec II	I	<1.5	-	<1.5	-	<1.5	-	10.8	0.4	11.0	<1	<1	<1	99
	O	<1.5	-	<1.5	-	2.30	0.08	30.2	1.2	32.5	<1	<1	7	92
Potokowa	I	<1.5	-	<1.5	-	5.0	0.2	51.6	2.1	56.6	<1	<1	10	88
	O	<1.5	-	<1.5	-	12.0	0.4	63.3	2.6	75.3	0	0	15	85
						Cd								
Nowiec II	I	0.006	0.000	0.005	0.000	0.001	0.000	0.008	0.000	0.019	30	25	4	41
	O	0.025	0.001	0.077	0.002	0.130	0.003	0.005	0.000	0.227	10	33	56	1
Potokowa	I	0.035	0.001	0.033	0.001	0.187	0.004	0.133	0.004	0.369	9	8	50	33
	O	0.047	0.001	0.041	0.001	0.267	0.006	0.196	0.006	0.558	9	7	48	36

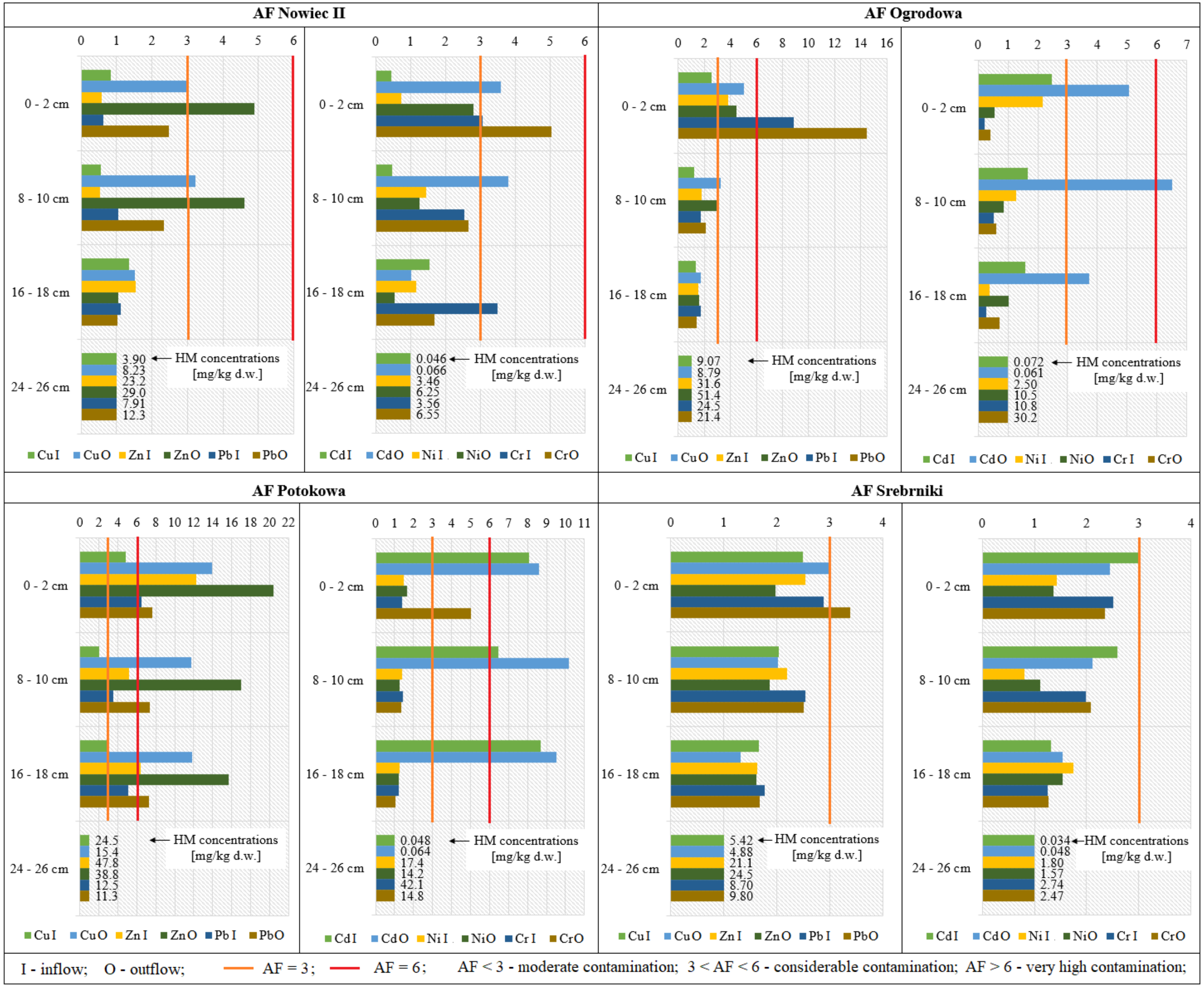

Figure Suplement 1 Anthropogenic Factor of vertical profile of sediments deposited in RTs on Strzyza Stream
